# Overexpression of Pyrabactin Resistance-Like Abscisic Acid Receptors Enhances Drought, Osmotic, and Cold Tolerance in Transgenic Poplars

**DOI:** 10.3389/fpls.2017.01752

**Published:** 2017-10-13

**Authors:** Jingling Yu, Haiman Ge, Xiaokun Wang, Renjie Tang, Yuan Wang, Fugeng Zhao, Wenzhi Lan, Sheng Luan, Lei Yang

**Affiliations:** ^1^State Key Laboratory for Pharmaceutical Biotechnology, NJU–NFU Joint Institute for Plant Molecular Biology, College of Life Sciences, Nanjing University, Nanjing, China; ^2^Department of Plant and Microbial Biology, University of California, Berkeley, Berkeley, CA, United States

**Keywords:** *Populus*, ABA receptor, drought, cold stress, osmotic stress, resistance

## Abstract

Abscisic acid (ABA) has been known participate in a wider range of adaptive responses to diverse environmental abiotic stresses such as drought, osmosis, and low temperatures. ABA signaling is initiated by its receptors PYR/PYL/RCARs, a type of soluble proteins with a conserved START domain which can bind ABA and trigger the downstream pathway. Previously, we discovered that poplar (*Populus trichocarpa*) genome encodes 14 PYR/PYL/RCAR orthologs (PtPYRLs), and two of them, PtPYRL1 and PtPYRL5 have been functionally characterized to positively regulate drought tolerance. However, the physiological function of these ABA receptors in poplar remains uncharacterized. Here, we generated transgenic poplar plants overexpressing PtPYRL1 and PtPYRL5 and found that they exhibited more vigorous growth and produced greater biomass when exposed to drought stress. The improved drought tolerance was positively correlated with the key physiological responses dictated by the ABA signaling pathway, including increase in stomatal closure and decrease in leaf water loss. Further analyses revealed that overexpression lines showed improved capacity in scavenging reactive oxygen species and enhanced the activation of antioxidant enzymes under drought stress. Moreover, overexpression of *PtPYRL1* or *PtPYRL5* significantly increased the poplar resistance to osmotic and cold stresses. In summary, our results suggest that constitutive expression of *PtPYRL1* and *PtPYRL5* significantly enhances the resistance to drought, osmotic and cold stresses by positively regulating ABA signaling in poplar.

## Introduction

Plants, as sessile organisms, have evolved sophisticated developmental and physiological strategies to adapt to the unfavorable and changing environments such as drought, high salinity, and temperature fluctuations. Drought has particularly been considered as one of the most serious natural hazards for agriculture due to the increasing water scarcity (Liu et al., 2015). Numerous studies showed that drought stress exerts many negative effects on plant growth, photosynthesis, biomass accumulation, and ecosystem carbon cycling (Liu et al., 2011; Zhou et al., 2015). Desiccation results in the production of reactive oxygen species (ROS), the production of which, in turn, serves as an important indicator to multiple abiotic stresses (Apel and Hirt, 2004; Contreras-Porcia et al., 2011). Normally, ROS are rapidly scavenged as a result of the activation of an efficient antioxidant system involved in various drought-induced signaling pathways, which are modulated by abscisic acid (ABA) (Jiang and Zhang, 2002; Contreras-Porcia et al., 2011).

The physiological functions of ABA has been extensively investigated in plants since it was identified in the 1960s as an endogenous plant hormone which regulates many essential processes, including seed germination, stomatal movement, plant development and adaptive responses, to multiple environmental stresses, such as drought, extreme temperatures, hyperosmolarity, and salinity (Finkelstein et al., 2002; Park et al., 2009). Many key components in ABA signaling pathway have been identified at the molecular level (Finkelstein et al., 2002), including ABA receptors (Shen et al., 2006; Liu et al., 2007; Pandey et al., 2009), the group A type 2 C protein phosphatases (PP2Cs) that negatively regulate ABA signaling at an early step in the pathway (Allen et al., 1999), the SnRK2 kinases that are positive regulators (Mustilli et al., 2002; Yoshida et al., 2002; Fujii et al., 2007), transcription factors (Seki et al., 2002; Himmelbach et al., 2003) and ion channels (Lee et al., 2009).

As the initial sensor of ABA signaling pathway, 14 genes designated as *Pyr1* and *Pyl1-Pyl13* (for *PYR1-Like*) have been identified in the *Arabidopsis* genome and encode proteins belonging to members of the cyclase subfamily of the START/Bet v I superfamily, which share a conserved hydrophobic ligand-binding pocket (Ma et al., 2009; Park et al., 2009). In presence of ABA, these proteins perceive ABA, then undergo conformation changes, and subsequently bind to clade A subfamily of PP2Cs, thus de-repressing the inactivation of the downstream SNF1-related protein kinase 2 (SnRK2) kinases. The PYR/PYL/RCAR-PP2C-SnRK2 signaling module is conserved across land plants.

Genetic evidence suggested that higher-order mutants lacking multiple ABA receptors in *Arabidopsis* (*Pyr1;Pyl1;Pyl2;Pyl4;Pyl5;Pyl8*) exhibit severe ABA insensitive phenotypes, establishing their critical physiological roles in ABA signaling (Gonzalez-Guzman et al., 2012). *PYL5* over-expression in *Arabidopsis* led to a globally enhanced response to ABA and enhanced drought resistance (Santiago et al., 2009). Constitutive overexpression of PYL8/RCAR3 confers ABA hypersensitivity in *Arabidopsis* seeds (Saavedra et al., 2010). Overexpression of *NtPYL4* in tobacco hairy roots resulted in a reprogramming of the cellular metabolism that is represented by a decreased alkaloid accumulation and conferred ABA sensitivity to the production of alkaloids (Lackman et al., 2011).

Tree species in the genus *Populus* spp., commonly known as poplars, aspens, and cottonwoods, are widespread in the northern hemisphere with nutrient-poor environments, and are increasingly important for bioenergy, wood products, and environmental services (Doty et al., 2016). Most of the poplar cultivation and distribution area are in the desolate lands on the earth where it is usually cold and dry in winter, causing the restriction of poplar forestry. Therefore, it is very critical to improve the resistance of poplars to abiotic stresses, including drought-resistance and cold-resistance. We have previously shown that PtPYRL1 and PtPYRL5 (poplar AtPYR1-like 1 and 5) physically interacted with PP2Cs, which interacted with SnRK2 kinase, suggesting they might act as the ABA receptors in mediating ABA signal transduction through phosphorylation and dephosphorylation (Yu et al., 2016). Furthermore, overexpression of *PtPYRL1* or *PtPYRL5* in *Arabidopsis* enhanced ABA sensitivity and drought-resistance. However, it is still unknown if they also play a similar function in poplars which protect against drought stress. Here, we report that the overexpression of *PtPYRL1* and *PtPYRL5* in poplar enhances resistance to drought, osmosis, and cold, the abiotic stresses that poplar frequently encounters. Our results also provide a potential biotechnological tool in engineering stress-resistant poplar cultivars.

## Materials and Methods

### Plant Materials and Growth Conditions

The sterile wild-type and transgenic hybrid poplar (*Populus davidiana* × *Populus bolleana*) were amplified by micropropagation with leaf bud explants and kept under a 16-h-light/8-h-dark photoperiod (120 μmol⋅m^-2^⋅s^-1^) at 21–24°C. The plantlets were sub-cultured onto fresh 1/2 Murashige and Skoog (MS) medium supplemented with 0.1 mg⋅L^-1^ naphthaleneacetic acid (NAA) and 1% (w/v) agar. Two-week-old wild type (WT) or transgenic hybrid poplars with new roots were transferred to 7.5 cm-width pots containing nutrient soil. The plants were grown in greenhouse under a 16-h-light/8-h-dark (120 μmol⋅m^-2^⋅s^-1^) at 21–24°C.

### Poplar Transformation

To construct the pCAMBIA1301S2-*PtPYRL1* (or *PtPYRL5*) plasmid, the entire coding region of *PtPYRL1* (or *PtPYRL5*) was amplified by PCR with *Xba I*-*Sal I* (or *BamH I*-*Sal I*) linker primers and cloned into modified pCAMBIA1301S2 with the 2×CaMV 35S promoter via the *Xba I-Sal I* (or *BamH I*-*Sal I*) site (**Supplementary Figure S1**). The hybrid poplars (*Populus davidiana × Populus bolleana*) were transformed using *Agrobacterium tumefaciens* (EHA105 strain) infection method. Briefly, leave explants excised from 1- to 2-month-old were inoculated in the EHA105 culture resuspended with liquid MS for 10 min and then were plated on solid MS medium containing 0.4 mg⋅L^-1^ 6-BA, 0.1 mg⋅L^-1^ NAA and 0.01 mg⋅L^-1^ TDZ. After 2 days of co-cultivation with *Agrobacterium*, the explants were transferred onto fresh MS medium containing 400 mg⋅L^-1^ timentin, and 10 mg⋅L^-1^ hygromycin for selective regeneration. When regenerated shoots reached 1 cm tall, they were excised and placed on rooting medium. Then, the rooted seedlings were transferred into soil and grown in the greenhouse. The presence of transgene was verified by PCR from genomic DNA, using primers specific for P35S and *PtPYRL1*-RT-R or *PtPYRL5*-RT-R.

### Histochemical GUS Analysis

Detection of β-glucuronidase (GUS) activity was performed as described by Jefferson et al. (1987) with some modifications: leaf explants were incubated in GUS assay buffer (50 mM sodium phosphate, pH 7.0, 0.1% [v/v] Triton X-100, 0.5 mM ferricyanide, 0.5 mM ferrocyanide, and 2 mM 5-bromo-4-chloro-3-indolyl-β-D-glucuronide) for 12 h at 37°C. Then the plant tissues were decolorized in 75% ethanol for three times. The samples were photographed after treatment.

### Quantitative Real-time PCR Analysis

Total RNA was extracted from various samples using TRIzol reagent (Invitrogen, Carlsbad, CA, United States), which was sequentially treated with DNase I (Invitrogen) and reverse transcribed by M-MLV reverse transcriptase (Promega). In semi-quantitative RT-PCR and qRT-PCR assays, the poplar elongation factor gene *EF1β* was used as an internal reference. qRT-PCR was performed with a CFX Connect Real-Time System (Bio-Rad). The relative expression of series indicated genes was calculated based on the comparative threshold cycle method using *EF1β* as a control and normalized to the WT hybrid poplars (under normal conditions). All primers used in this study were listed in Supplementary Table S1.

### Drought–Rehydration Experiments

Two-week-old WT or transgenic poplars were transplanted to each 7.5 cm-width pot containing 50 g nutrient soil for greenhouse cultivation. We chose the 2-month-old WT or transgenic poplar seedlings with the same height to perform the drought and rehydration experiments. As the control, half of WT and transgenic poplar seedlings were normally watered, and the rest of these seedlings were not watered. After 1 week, the plantlets were watered again. After 3 days, the re-watered plants were photographed.

### Water Potential Determination

Leaf water potential was measured on the same location of the blade with a WP4-T Dew point PotentiaMeter (Decagon Devices, Inc., United States). Five individual plants from WT or different transgenic poplars were measured after drought treatment for 5 days.

### Measurement of Contents of H_2_O_2,_ MDA, and Proline

H_2_O_2_ in fresh leaves was analyzed using the method reported by Hu et al. (2012). The absorbance was recorded at 390 nm with the spectrophotometer (Biomate 3S, Thermo). Malondialdehyde (MDA) content was quantified using the method reported by Heath and Packer (1968), which is related with the level of lipid peroxidation in the leaves. The absorbance was read at 532 and 600 nm by the spectrophotometer (Biomate 3S, Thermo) with thiobarbituric acid (1%) in 20% trichloroacetic acid as control. The Proline content in leaves was quantified using the method by Bates et al. (1973). The absorbance was measured spectrophotometrically (Biomate 3S, Thermo) at 520 nm and toluene was used as blank.

### Extraction and Assay of Antioxidant Enzymes

Fresh leaves (0.5 g/sample) were homogenized in presence of 100 mM Tris-HCl (5.0 ml, pH 7.5), 3.0 mM β-mercaptoethanol, 1.0 mM EDTA (ethylenediaminetetraacetic acid), and 1.5% polyvinylpyrrolidone-40. The mixture was better supplemented with serine and cysteine proteinase inhibitors [1.0 mM phenylmethanesulfonyl fluoride (PMSF) + 1.0 μg⋅mL^-1^ aprotinin]. The homogenate was centrifuged at 10,000 × *g* for 15 min (4°C) after the filtration through cheese cloth. The supernatants were collected and served as the crude enzyme for determination of SOD (EC1.15.1.1), CAT (EC1.11.1.6), and POD (EC 1.11.1.7) activities. For the determination of APX activity, leaf sample was separately grounded in a homogenizing medium supplemented with 2.0 mM ascorbic acid (AsA) to maintain the enzyme stability. SOD activity was analyzed after the photoreduction of nitroblue tetrazolium (NBT) according to the method of Giannopolitis and Ries (1977). The absorbance was recorded spectrophotometrically (Biomate 3S, Thermo) at 560 nm. One unit of SOD is the quantity of protein that hampers 50% photoreduction of NBT and the activity was expressed as enzyme unit (EU)⋅mg^-1^ protein. The method of Chance and Maehly (1955) was employed to analyze CAT activity. The absorbance was read at 240 nm through a UV-Visible spectrophotometer (Biomate 3S, Thermo) and EU⋅mg^-1^ protein represented the CAT activity. The method of Nakano and Asada (1981) was used for the APX activity (EC1.11.1.11) measurement. The absorbance was recorded at 290 nm each 30 s for 3 min spectrophotometrically (Biomate 3S, Thermo). The POD activity was determined by examining the absorbance of reaction buffer at 420 nm based on guaiacol oxidation (Maehly and Chance, 1954). APX activity was calculated by the consumption rate of ASC using the ASC extinction molar coefficient (*e* = 2.8 mM^-1^⋅cm^-1^). APX activity was expressed with EU⋅mg^-1^ protein. One unit of APX is the quantity of protein used to break down 1.0 μmol of substrate per min at 25°C. The activity of GR (EC 1.6.4.2) and GPX (EC 1.11.1.9) was determined using GR Assay Kit (S0055, Beyotime, China) and Total GPX Assay Kit (S0058, Beyotime, China), respectively, according to the manufacturer’s instructions. The absorbance was read at 340 nm with spectrophotometer (Biomate 3S, Thermo). GR activity was expressed as μmol NADPH oxidized min^-1^ (EU⋅mg^-1^ protein) (Carlberg and Mannervik, 1985). The GPX activity was calculated by measuring the reduction of NADPH to NADP^+^ at 340 nm of absorbance.

### Analysis of Relative Water Content (RWC)

The extent of desiccation in WT and transgenic poplars aboveground part was indicated with RWC (%) following the formula RWC % = (*W*_de_ – *W*_dr_)/(*W*_f_ - *W*_dr_) × 100, where *W*_f_ is the wet weight of fully hydrated aboveground part, *W*_de_ is the dehydrated weight after desiccation for a period of time, and *W*_dr_ is the dry weight determined after 48 h of drying at 80°C. Through this, the RWC reflects the extent of desiccation, with a fully hydrated shoot having a RWC of 100% and a fully dehydrated shoot having a RWC close to 0%. A lower RWC indicates higher desiccation.

### Stomatal Aperture Measurements

To measure stomatal aperture in response to ABA, epidermal peels of the leaves in the same location were floated on a stomatal opening medium containing 50 mM KCl, 10 mM MES-KOH (pH 6.15), and 0.1 mM CaCl_2_ and incubated in a growth chamber under white light (150 μmol⋅m^-2^⋅s^-1^) for 2 h. The epidermal strips were transferred to the opening medium with 0 or 20 μM ABA and incubated for a further 2 h before stomatal apertures were measured.

### Hyperosmotic Stress Treatment

The 5 cm apical shoot segments of WT and transgenic poplars were transferred into the 1/2 solid Murashige and Skoog (MS) medium containing 0.1 mg⋅L^-1^ naphthaleneacetic acid and 1% (w/v) agar supplemented with 0, 200, or 300 mM mannitol. The photos were taken at the 30th day after mannitol treatment. The experiments were repeated three times.

### H_2_O_2_ Staining

H_2_O_2_ in leaves was visualized by 3,3-diaminobenzidine (DAB). *Populus* leaves were cut at the leaf base and infiltrated in 1 mg/mL DAB solution (50 mM Tris-HAC, pH 5.0) for 2–8 h. Samples were then decolorized in 95% ethanol at 80°C for 2 h. Brown flecks indicate the accumulation of H_2_O_2_ (Yang et al., 2004). The leaves were observed and photographed under stereomicroscope.

### Statistical Analysis

The experiments were repeated three times and all comparisons of average values were analyzed using one-way ANOVA test. *Post hoc* comparisons were performed by applying least significant difference test. Significant differences were indicated with the threshold of *P* < 0.05.

## Results

### Molecular Characterization of the Transgenic *PtPYRLs* Poplar Plants

**FIGURE 1:**
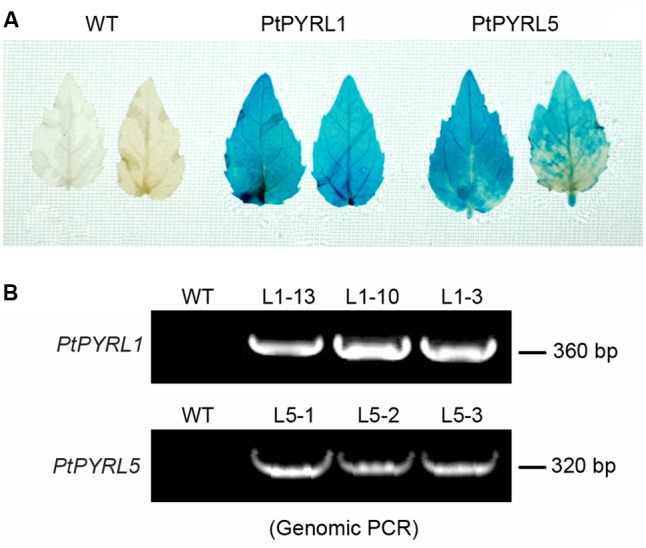
Identification of transgenic poplar plants. **(A)** Histochemical analysis of GUS histochemical in *PtPYRL1* and *PtPYRL5* transgenic poplar leaves. **(B)** PCR analysis of genomic DNA from different independently regenerated hygromycin resistant lines using 35S and *PtPYRL1* and *PtPYRL5* specific primers.

**FIGURE 2:**
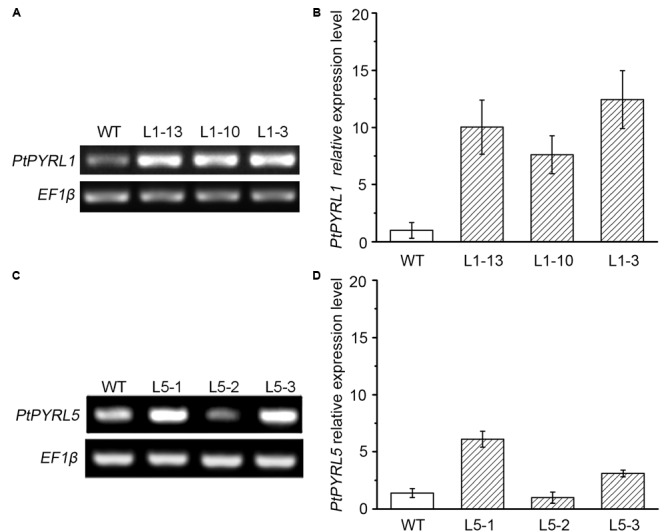
Expression of *PtPYRL1* or *PtPYRL5* in wild type (WT) and transgenic hybrid poplars. Expression of *PtPYRL1* **(A)** or *PtPYRL5* **(C)** in 2-month-old WT and transgenic hybrid poplars (L1-13, L1-10, and L1-3) grown at normal medium. The level was detected using gene-specific primers after 26 PCR cycles. *EF1β* was used as endogenous control in all cDNA samples. Relative levels of *PtPYRL1* transcripts **(B)** or *PtPYRL5* transcripts **(D)** in three transgenic lines by qRT-PCR. Total RNA was isolated from 2-month-old leaves grown at normal medium. The relative expression level was calculated as the ratio of *PtPYRL* level to endogenous control *EF1β* level.

To obtain transgenic poplar lines that overexpress the ABA receptors *PtPYRL1* and *PtPYRL5* genes under the control of the CaMV 35S promoter were introduced into the leaf explants from a hybrid poplar cultivar (*Populus davidiana* × *Populus bolleana*), respectively. After cultured in the medium containing timentin and hygromycin, shoots were regenerated from some transfected explants (**Supplementary Figure S2A**). Partial regenerated shoots formed roots when they were placed on rooting medium (**Supplementary Figure S2B**), and these rooted plantlets were selected to analyze *35S-PtPYRL1* or *35S-PtPYRL5* insertion by PCR analysis of genomic DNA. A reporter gene encoding GUS was co-transferred into the leaf explants via the same vector, to authenticate the transgene expression in the putative transgenic lines. The GUS staining analysis revealed that all rooted seedlings expressed GUS activity properly (**Figure 1A**), confirming the integration and expression of the transgene in the genome of the transformants (**Figure 1B**). More than 10 independent lines were obtained for each of transgenes, including the lines L1-3, L1-10, and L1-13 expressing *PtPYRL1*, and the lines L5-1, L5-2, and L5-3 expressing *PtPYRL5* (**Figure 1B**). After the rooted seedlings were transferred into soil, the survival seedlings were selected for further analysis of the differential expression levels of *PtPYRLs* by RT-PCR (**Figures 2A,C**) and qRT-PCR (**Figures 2B,D**). Among of them, Lines L1-3, L1-10, and L1-13 had over eightfold increment of the relative expression level of *PtPYRL1* than in WT (**Figure 2B**), and lines L5-1 and L5-3 had over threefold increment of the relative expression level of *PtPYRL5* compared with WT (**Figure 2D**). Thus, two lines with high level of *PtPYRL1* (L1-3 and L1-10) or of *PtPYRL5* (L5-1 and L5-3) were used for subsequent physiological analysis.

### Overexpression of Poplar ABA Receptors Enhanced Drought Tolerance in Transgenic Poplar Plants

**FIGURE 3:**
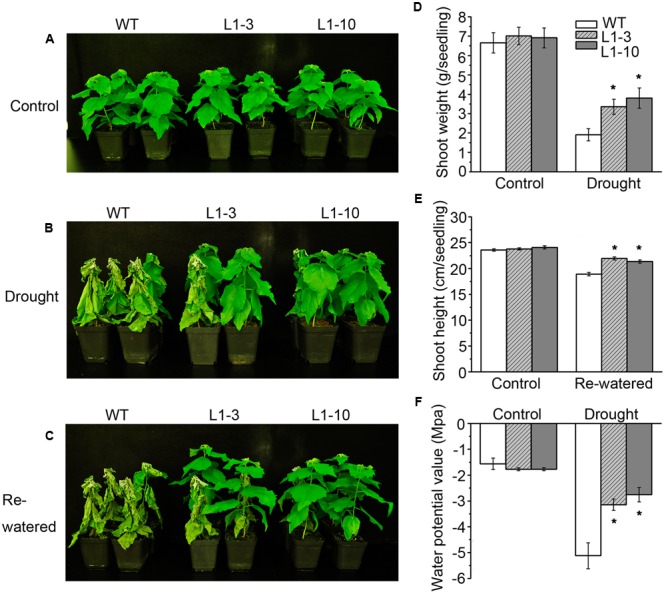
Overexpression of poplar ABA receptor *PtPYRL1* in poplars enhanced drought-stress resistance. **(A)** Two-month-old WT (non-transgenic hybrid poplars) and transgenic poplars (L1-3 and L1-10) were cultured in the greenhouse with normal watering. **(B)** WT and transgenic hybrid poplars were not watered for 5 days. **(C)** After drought, WT and transgenic hybrid poplars were then re-watered for 3 days. The shoot weight after drought stress treatment **(D)**, shoot height after re-watered for 3 days **(E)**, water potential value **(F)** of WT and transgenic hybrid poplars were measured. Values are means ± standard deviation (SD) (one-way ANOVA test; ^∗^*P* < 0.05 as compared to WT).

To investigate the role of *PtPYRLs* in drought tolerance, 2-month-old wild-type, *PtPYRL1* transgenic plants (L1-3 and L1-10), and *PtPYRL5* transgenic plants (L5-1 and L5-3) were subjected to water withhold for 5 days and then re-watered for 3 days. After 5 days of water deprivation, wild-type plants began to wilt, while most of transgenic plants remained fresh and alive (**Figure 3B**), similar to those grown under the watering condition (**Figure 3A**). When plants were re-watered for 3 days, WT became wilted permanently and eventually died, whereas transgenic plants showed less damaged and most of them recovered growth (**Figure 3C**). Similarly, *PtPYRL5* transgenic plants [L5-1 and L5-3 and another two *PtPYRL1* transgenic lines (L1-8 and L1-5)] displayed a healthier growth compared with the WT after rewatering (**Supplementary Figures S3A–C**). Furthermore, transgenic poplars overexpressing *PtPYRL1* or *PtPYRL5* showed more shoot biomass (**Figure 3D** and **Supplementary Figures S3D, S4D**) and shoot height (**Figure 3E** and **Supplementary Figures S3E, S4E**) than WT. They also had higher leaf water potential Ψ than WT (**Figure 3F** and **Supplementary Figures S3F, S4F**), indicating the leaves of transgenic lines retained more water during the stress.

**FIGURE 4:**
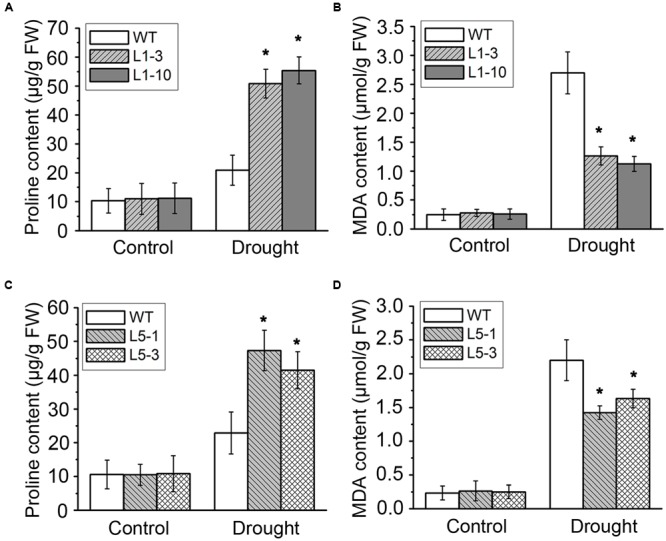
Proline **(A,C)** and MDA **(B,D)** contents were measured in WT and PtPYRL1 or PtPYRL5 transgenic hybrid poplars after drought treatment. Values are means ± SD (one-way ANOVA test; ^∗^*P* < 0.05 as compared to WT).

**FIGURE 5:**
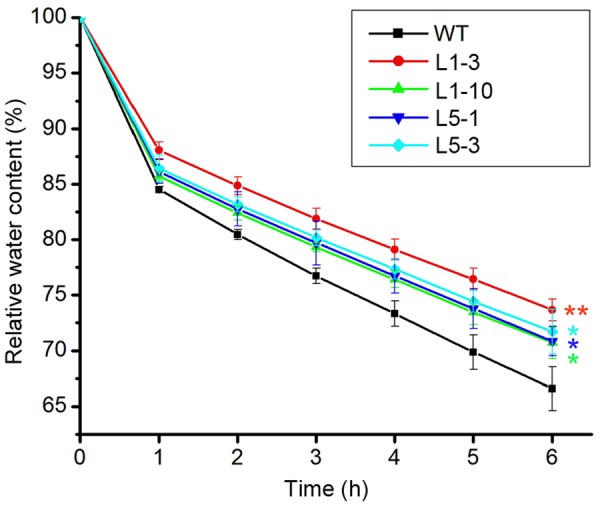
Relative water content (RWC) of WT and *PtPYRL1, PtPYRL5* overexpressing poplars after exposure in the air. RWC during a 6-h period from the detached shoot of the WT, *PtPYRL1*- and *PtPYRL5*-overexpressing hybrid poplars. Each value is the mean ± SD of three biological determinations. One-way ANOVA test was used to compare the RWC of each transgenic poplar with those of the WT (with significant differences at ^∗^*P* < 0.05 as compared to WT; ^∗∗^*P* < 0.01 as compared to WT).

Drought stress severely impairs the cellular lipid structure and function in tree species (Štajner et al., 2011), and thus the corresponding products proline and MDA were measured in these poplars. Though the proline and MDA contents in both WT and transgenic poplars were increased after drought stress treatment, the transgenic poplars contained higher proline level (**Figures 4A,C**) and less MDA content (**Figures 4B,D**) than WT. It is noteworthy that the changes of biomass, water potential, proline, and MDA in the transgenic *PtPYRL5* lines (L5-1 and L5-3) were less significant than the transgenic *PtPYRL1* lines (L1-3 and L1-10), suggesting that PtPYRL1 may be more potent than PtPYL5 in inducing the downstream protective responses. Moreover, relative water content (RWC) of detached leaves was higher from transgenic *PtPYRL1* lines (L1-3 and L1-10) than from WT plants after exposed in air for 6 h (**Figure 5**). Taken together, these results indicated that overexpression of *PtPYRL1* or *PtPYRL5* in poplars enhanced the resistance to drought stress, probably by reducing water loss rate.

### Effects of *PtPYRLs* Overexpression on Antioxidant Metabolism in Poplars under Drought Stress

**FIGURE 6:**
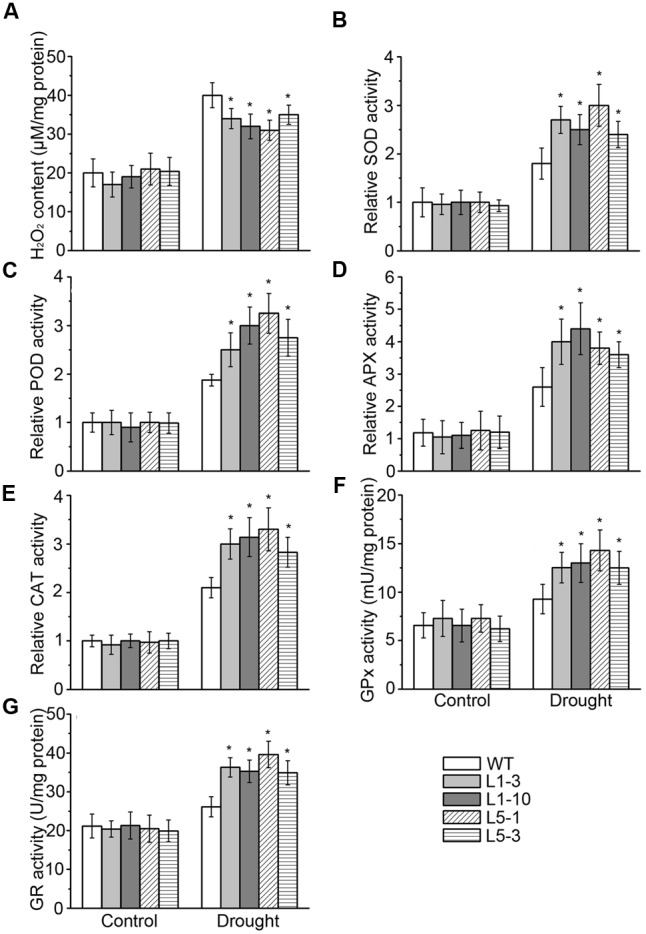
H_2_O_2_ content **(A)**, SOD **(B)**, POD **(C)**, APX **(D)**, CAT **(E)**, GPx **(F)**, and GR **(G)** activities assay of WT, *PtPYRL1*, and *PtPYRL5* overexpressing poplars under drought conditions. 2-month-old WT and transgenic hybrid poplars (L1-3, L1-10, L5-1, and L5-3) were watered regularly, drought stress was performed on these poplars withholding water for additional 5 days, and the leaves were collected. Values for enzyme activities such as SOD, POD, APX, and CAT were normalized to those for the WT hybrid poplars grown under well-watered conditions, which were set at one. Error bars show standard deviations (*n* = 12, one-way ANOVA test; ^∗^*P* < 0.05 as compared to WT).

Lipid hydroperoxidation is considered as the biochemical indicator of cellular oxidative damage, which is induced by excessive accumulation of the reactive oxidative species (e.g., superoxide and H_2_O_2_) in plant cells (Yoshimura et al., 2004; Sochor et al., 2012). Therefore, we measured H_2_O_2_ content in the leaves of WT and transgenic plants. In parallel with the increase in MDA contents indicated in **Figure 4**, cellular H_2_O_2_ levels were also increased by drought stress in the WT and transgenic *PtPYRL1* or *PtPYRL5* plants. However, H_2_O_2_ level was significantly lower in these transgenic hybrid poplars than that in the WT leaves (**Figure 6A**). Correspondingly, the activity of the key enzymes controlling ROS scavenging, including superoxide dismutase (SOD), peroxidase (POD), ascorbate peroxidases (APX), and catalase (CAT), were analyzed in these poplars under drought stress. Indeed, the activity of SOD, POD, APX, and CAT were remarkably enhanced in transgenic hybrid poplars compared with the WT (**Figures 6B–E**). Besides these antioxidant enzymes, plants have several non-enzymatic antioxidants important for redox equilibrium, such as glutathione (GSH) (Cao et al., 2009). GSH content is controlled by glutathione reductase (GR), which has an important role in maintaining the level of glutathione, and by glutathione peroxidases (GPx), which can catalyze the reduction of lipid peroxide through glutathione. Upon drought stress, transgenic hybrid poplars had higher activity of GR and GPx than WT (**Figures 6F,G**). These results indicated that overexpression of *PtPYRL1* and *PtPYRL5* resulted in greater stimulation of the activity of enzymes responsible for ROS and GSH metabolism in poplars upon drought stress.

### Overexpression of *PtPYRL1* or *PtPYRL5* Accelerated Stomatal Closure Induced by ABA

**FIGURE 7:**
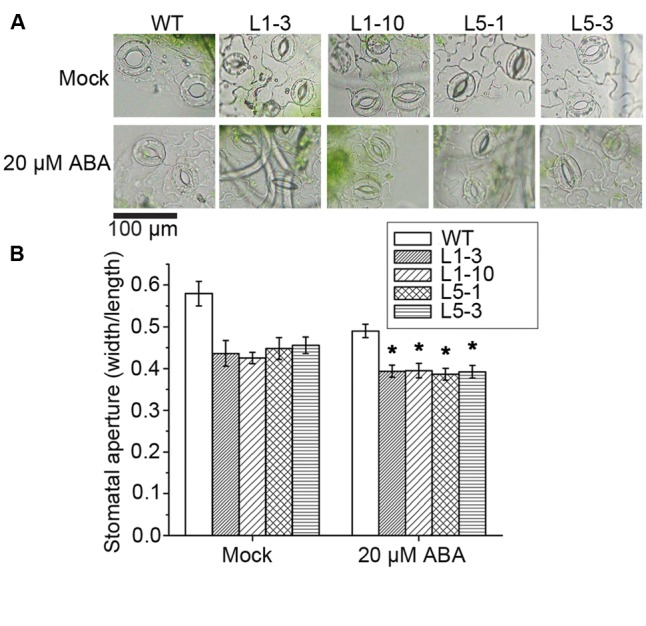
Stomatal movement in WT and *PtPYRL1, PtPYRL5* overexpressing hybrid poplars under ABA treatment. **(A)** The representative images of stomata shape of WT and transgenic seedlings in the presence or absence of ABA. The 2-month-old WT and transgenic seedlings L1-3, L1-10, L5-1, and L5-3 were treated with stomata opening buffer (mock) (upper), or with 20 μM ABA (lower). Pictures were taken 2 h after ABA treatment in Olympus BX51 microscope in 40× magnification. Scale bar is 100 μm. **(B)** Stomatal aperture of WT and transgenic seedlings at 2 h after treatment with stomatal opening buffer (mock) or with 20 μM ABA. Stomatal aperture was calculated as the ratio of stomatal width to length. About 200 stomata were analyzed for each genotype. Bars represent mean ± SEM of three independent experiments and significant differences are indicated as ^∗^*P* < 0.05.

The stomatal movement is one of the most important responses in plants under drought stress, and stomatal behavior is highly controlled by ABA. ABA was thus applied to assess the potential difference in stomatal movement between the WT and the transgenic *PtPYRLs* poplars. Without any treatment, even though overexpressing lines have more closed stomata, the difference was not significant, as shown by ANOVA test. With 20 μM ABA treatment, a significant decrease in stomatal aperture was found for both WT and the transgenic *PtPYRLs* poplars (**Figure 7A**). There was a significant difference between WT-mock and WT-ABA using ANOVA test. Stomatal apertures in the *PtPYRL1-* and *PtPYRL5-*overexpressing lines L1-3, L1-10, L5-1, and L5-3 reduced to 0.394, 0.395, 0.387, and 0.392, respectively, while stomatal aperture of WT became 0.490 (**Figure 7B**), which was significantly larger than that of *PtPYRL1-* and *PtPYRL5-*overexpressing lines. These results indicate that the overexpression of *PtPYRL1* and *PtPYRL5* enhanced poplar stomatal closure in response to ABA, which might be the cause of the enhanced drought tolerance of transgenic plants.

### Overexpression of *PtPYRL1* or *PtPYRL5* Enhanced Osmotic Stress Resistance

**FIGURE 8:**
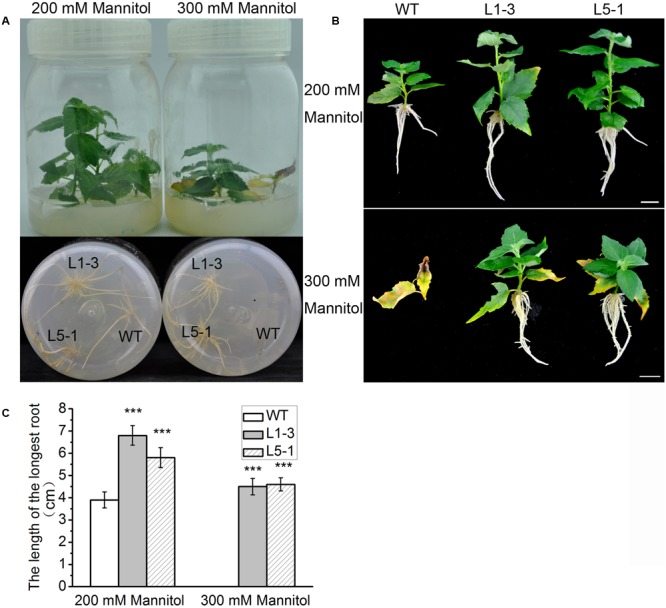
Overexpression of *PtPYRL1/5* in hybrid poplar improved resistance to hyperosmotic stress. **(A)** Short shoot segments (upper) and new fibrous roots (down) grown for 5 days on 1/2 MS medium supplemented with mannitol (200 and 300 mM). Under 200 and 300 mM mannitol treatment, in comparison to the transgenic poplars, the damage to apical leaves bud and new roots of WT is more serious. **(B)** Outgrowth of fibrous roots of shoot segments from poplars overexpressing *PtPYRL1/5* (L1-3 and L5-1) is not inhibited by 300 mM mannitol, however, outgrowth of fibrous roots of shoot segments from WT poplars is inhibited by 300 mM mannitol. Bars represent 1 cm. **(C)** The length of the fibrous root of shoot segments in **(B)**. Values are the mean ± SD of three independent experiments (*n* = 10 shoots segments, ^∗∗∗^*P* < 0.001 as compared to WT).

Osmotic damage is a major consequence of drought stress in plants, and ABA signaling contributes to damage reduction (Skirycz and Inzé, 2010). Overexpression of *PtPYRL1* or *PtPYRL5* enhanced resistance to water deficit (**Figure 3** and **Supplementary Figures S3, S4**), which suggested a possible role of *PtPYRL1* or *PtPYRL5* in high osmotic stress resistance. To confirm this possibility, wild-type and transgenic shoots were cultured on 1/2 MS supplemented with 200 or 300 mM mannitol. The supplementation of mannitol inhibited the growth of WT and transgenic poplars (**Figure 8A**). At the presence of 300 mM mannitol, the WT plants did not even survive and their leaves show yellowish and withered, while young leaves of transgenic plants overexpressing *PtPYRL1* or *PtPYRL5* kept green and some old leaves displayed yellowish at leaf edge (**Figures 8A,B**). In addition, unlike WT plants, all transgenic plants generated new roots successfully in 1/2 MS medium supplemented with 300 mM mannitol (**Figures 8A,B**). According to the statistical analysis shown in **Figure 8C**, transgenic plants overexpressing PtPYRL1 or PtPYRL5 had roots with an average length of around 4.5 cm, while the WT did not have visible roots. These results indicated that the transgenic poplar overexpressing *PtPYRL1* or *PtPYRL5* improved the resistance to hyperosmotic stress.

### Overexpression of *PtPYRL1* or *PtPYRL5* Reduced the Injury Induced by Low Temperature

**FIGURE 9:**
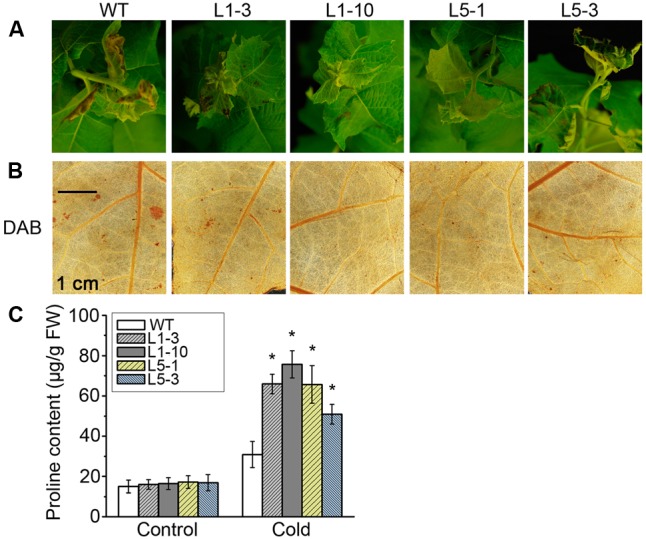
Cell viability, contents of hydrogen peroxide, and osmolytes in leaves of WT and *PtPYRL1, PtPYRL5*- overexpressing poplars under low temperature treatments. **(A)** The phenotype of WT and transgenic hybrid poplars (L1-3, L1-10, L5-1, and L5-3) under cold stress treatments. Hydrogen peroxide in leaves were visualized by DAB **(B)**, respectively. **(C)** Proline content of WT and *PtPYRL1-* or *PtPYRL5-* overexpressing hybrid poplars. Values are means ± SD of three independent experiments (^∗^*P* < 0.05 as compared to WT).

We also subjected WT and transgenic *PtPYRL1* or *PtPYRL5* poplars to low temperature which is a common stress in poplars growth and found that *PtPYRL1* and *PtPYRL5* transgenic poplars displayed the increased resistance under chilling stress (**Figure 9**). After 4°C treatment for 5 days, the apical young leaves of WT were damaged more severely with leaf wilting and necrosis than that of transgenic poplars (**Figure 9A**). Furthermore, the leaves of WT had a higher accumulation of hydrogen peroxide visualized by 3,3-diaminobenzidine (DAB) (**Figure 9B**). Meanwhile, the leaves of WT contained less proline accumulation, a key anti-freeze component (**Figure 9C**). These results suggested that overexpression of *PtPYRL1* or *PtPYRL5* alleviated the injury induced by low temperature in poplars.

## Discussion

As initial factors in triggering ABA signaling, the physiological function of PYR/PYL/RCARs family is supposed to be critical in the evolution from aquatic to terrestrial plants. *PYR/PYL/RCAR* genes have been reported to be present as 13–14 members in the genome of *Arabidopsis*, rice and *Populus* (Ma et al., 2009; Tian et al., 2015; Yu et al., 2016). As a class of ubiquitous soluble protein, the *PYR/PYL/RCARs* family plays a critical role in ABA response and signal transduction in plants. Overexpression of *Arabidopsis PYR/PYL/RCAR* receptors is known to enhance ABA response and plant drought tolerance (Santiago et al., 2009; Saavedra et al., 2010; Pizzio et al., 2013). Our previous study showed the transgenic *Arabidopsis* overexpressing *PtPYRL1* or *PtPYRL5* were both hypersensitive to ABA and enhanced drought resistance (Yu et al., 2016). In the present study, we further provide evidence that these two PtPYRL*s* are very important in poplars to confer tolerance to diverse environmental abiotic stresses, including drought, hyper-osmosis, and low temperature.

The *PYR/PYL/RCAR* genes have been shown to enhance drought tolerance in *Arabidopsis*, rice, and tomato (González-Guzmán et al., 2014; Kim et al., 2014; Zhao et al., 2016). But the evidence of functional studies in the woody plants are still lacking. In our study, hybrid poplar *Populus davidiana* × *Populus bolleana* was transformed with the *PtPYRL1* or *PtPYRL5* gene and WT hybrid poplar served as control. We did not observe any phenotypic changes in *PtPYRL1/5* overexpression transgenic poplars under normal growth conditions. Compared with WT poplars, overexpression of *PtPYRL1* or *PtPYRL5* enhanced drought tolerance. The shoot weight, leaf water potential, and proline concentration were obviously increased, and MDA content was markedly reduced in transgenic poplars under drought condition, resulted in rapid recovery with higher shoot height after drought and re-watering treatment. Low water availability in the dry soil limited evaporation and made water potential in the cell drop, these changes were associated with reduction in the leaf RWC. The overexpression of *PtPYRL1* or *PtPYRL5* in poplars showed larger leaf water potential (Ψ) after drought treatment, probably due to increased water retention capacity of the cells, lower water loss rate as well as more adaptive stomatal movement during the stress (Leung and Giraudat, 1998; Zhu, 2002; Souza et al., 2004; Kwak et al., 2008). As showed in **Figure 7**, exogenous ABA promoted the stomata closure, *PtPYRL1* or *PtPYRL5* overexpressing transgenic poplars were more sensitive to ABA. Hereafter, recovery will be described as the visual evidence of new above ground development and growth, according to previous studies (Brodribb and Cochard, 2009; Petrov et al., 2015), which defined the process of recovery as a process of reactivated physiological processes and meristematic activity. *PtPYRL1* or *PtPYRL5* overexpressing transgenic poplars were taller and better recovered than WT poplars after drought and re-watering treatment (**Figure 3E**). Our findings supported the earlier finding that drought causes hydraulic restriction, with the subsequent development of high tension in the xylem water column and the closure of stomata (Tyree and Sperry, 1988). Larger aboveground biomass indicated better survival in the drought stressed *PtPYRL1* or *PtPYRL5* overexpressing transgenic poplars, while more proline accumulation resulted in the better water status maintenance (Bajji et al., 2001) and less MDA concentration led to better protection of the cell membrane structures in the drought treated *PtPYRL1* or *PtPYRL5* overexpressing transgenic poplars. The results presented here indicated that PtPYRL1/5 positively regulates ABA-related response to drought stress.

Drought commonly results in oxidative stress due to the over-production and over-accumulation of ROS derived from inefficient dissipation of excessive excitation energy (Shanker et al., 2014). The over-accumulation of ROS may lead to biochemical disruption of membranes and result in mortality (Suzuki et al., 2012; Petrov et al., 2015). ROS scavenging systems of plants detoxify ROS to minimize and/or prevent oxidative damage in cells by increasing the activity of ROS scavenging enzymes such as SOD, CAT, APX, POD, GR, and GPx (Gill and Tuteja, 2010). In the present study, the content of H_2_O_2_ was significantly lower in the *PtPYRL1*- and *PtPYRL5*-overexpressing poplars than in WT under drought stress (**Figure 6A**). Consistent with this phenomenon, all of the measured antioxidant enzyme activities showed notably increased in transgenic poplars after drought treatment (**Figures 6B–G**). It was proposed that the antioxidant protection is related to higher leaf water potential Ψ (**Figure 3F** and **Supplementary Figures S3F, S4F**) (Menconi et al., 1995). These results demonstrated that overexpression of *PtPYRL1* or *PtPYRL5* increased the antioxidant enzyme activities protecting transgenic poplars against the oxidative damage.

Consistently, osmotic stress significantly suppressed wild-type poplars roots development and leaf growth compared with *PtPYRL1* or *PtPYRL5* overexpressing poplars (**Figure 8**). The increased root growth rate of transgenic poplars (**Figure 8C**) has to be coupled with a mechanism that alleviates the physiological growth retardation consequences of the osmotic stress. In *PtPYRL1* or *PtPYRL5* overexpressing poplars, roots had increased the low water availability, as their rapid root growth resulted in more water absorption. Since the osmotic stress has been suggested to increase the ABA levels in plant cells (Zeevaart and Creelman, 1998), transgenic poplars might perceive ABA more rapidly and might trigger more efficiently the downstream responses. The phenotype of transgenic poplars under osmotic stress indicated that *PtPYRL1* or *PtPYRL5* increased the tolerance to hyperosmotic stress.

Another common environmental stress, a low temperature, severely inhibits plant growth and development. A recent study illustrated that co-overexpression of two genes, a bZIP transcription factor (*OsbZIP46CA1*) and a protein kinase (*SAPK6*) involved in the ABA signaling pathway, showed improved tolerance to heat and cold stresses in rice (Chang et al., 2017). In order to enhance plant adaptability to low temperature, lipids, amino acid, membrane components, and other molecules in the cell are produced to promote cell membrane fluidity and structural rearrangement (Maruyama et al., 2014; Wu et al., 2016). Balance of the cellular ROS homeostasis also contributes to the tolerance to temperature stresses (Wang et al., 2017). Our results showed that the *PtPYRL1* or *PtPYRL5* overexpressing poplars enhanced low temperature stress tolerance (**Figure 9**), associated with more efficient ROS scavenging (**Figure 9B**) and more proline accumulation to maintain membrane integrity (**Figure 9C**). On the other hand, it is well known that phytohormone ABA activates a cascade of downstream signaling events in response to cold exposure (Knight et al., 2004). Further studies are required to investigate how PtPYRL1 and PtPYRL5 mediates adaptive responses to cold at the molecular level. Moreover, though our data showed that both ABA receptors are over-expressed in transgenic poplar plants at the mRNA level, it would be interesting to test their protein accumulation in future studies, since it has been suggested that PYR/PYL receptors undergo regulation at protein level by 26S proteasome pathway in *Arabidopsis* (Bueso et al., 2014; Irigoyen et al., 2014).

*Populus* is a perennial woody model plant and also economically important tree. Most *Populus* are sensitive to environmental factors, which considerably affects their productivities. As we know, arid and semi-arid regions account for approximately 30% of the worldwide area (Sivakumar et al., 2005). Meanwhile, the climate in poplars cultivated land of China is mostly dry and frigid. Therefore, breeding high drought- and cold-tolerant poplar cultivars is very necessary for improving land use efficiency and poplar forestry development. In our study, transgenic poplar overexpressing *PtPYRL1* or *PtPYRL5* was found to be significantly more drought and chilling tolerant than WT plants. We hope that the transgenic poplars generated in this study can be used for cultivating in cold as well as arid and semi-arid areas.

## Author Contributions

LY, RT, and SL designed the study. LY, WL, HG, and JY revised the manuscript critically. LY, JY, RT, YW, XW, and FZ performed experiments, analyzed and interpreted the data, and wrote the manuscript.

## Conflict of Interest Statement

The authors declare that the research was conducted in the absence of any commercial or financial relationships that could be construed as a potential conflict of interest.

## Supplementary Material

FIGURE S1 The pCAMBIA1301S2-*PtPYRL1* (or *PtPYRL5*) Vector map.

FIGURE S2 Transformants selection. **(A)** Adventitious bud regenerated from selection medium. **(B)** Transgenic shoots formed roots on selection medium.

FIGURE S3 Overexpression of poplar ABA receptor *PtPYRL5* in poplars enhanced drought-stress resistance. **(A)** 2-month-old WT (non-transgenic hybrid poplars) and transgenic poplars (L5-1 and L5-3) were cultured in the greenhouse with normal watering. **(B)** WT and transgenic hybrid poplars were not watered for 5 days. **(C)** After drought, WT and transgenic hybrid poplars were then re-watered for 3 days. The shoot weight after drought stress treatment **(D)**, shoot height after re-watered for 3 days **(E)**, water potential value **(F)** of WT and transgenic hybrid poplars were measured. Values are means ± SD (one-way ANOVA test; ^∗^*P* < 0.05 as compared to WT).

FIGURE S4 Overexpression of poplar ABA receptor PtPYRL1 in poplars enhanced drought-stress resistance. **(A)** 2-month-old WT (non-transgenic hybrid poplars) and transgenic poplars (L1-8 and L1-5) were cultured in the greenhouse with normal watering. **(B)** WT and transgenic hybrid poplars were not watered for 5 days. **(C)** After drought, WT and transgenic hybrid poplars were then re-watered for 3 days. The shoot weight after drought stress treatment **(D)**, shoot height after re-watered for 3 days **(E)**, water potential value **(F)**, proline content **(G)** and MDA content **(H)** of WT and transgenic hybrid poplars were measured. Values are means ± SD (*n* = 18, three independent experiments, one-way ANOVA test; ^∗^*P* < 0.05).

## References

[B1] Allen G. J. Kuchitsu K. Chu S. P. Murata Y. Schroeder J. I. (1999). *Arabidopsis abi1-1* and *abi2-1* phosphatase mutations reduce abscisic acid-induced cytoplasmic calcium rises in guard cells. *Plant Cell* 11 1785–1798. 10.2307/3871054 10488243 PMC144302

[B2] Apel K. Hirt H. (2004). Reactive oxygen species: metabolism, oxidative stress, and signal transduction. *Annu. Rev. Plant Biol.* 55 373–399. 10.1146/annurev.arplant.55.031903.141701 15377225

[B3] Bajji M. Kinet J.-M. Lutts S. (2001). The use of the electrolyte leakage method for assessing cell membrane stability as a water stress tolerance test in durum wheat. *Plant Growth Regul.* 36 61–70. 10.1023/A:1014732714549

[B4] Bates T. R. Rosenberg H. A. Tembo A. V. (1973). Inconsistencies in rationale underlying official USP dissolution rate specifications for nitrofurantoin. *J. Pharm. Sci.* 62 2057–2058. 10.1002/jps.2600621241 4762187

[B5] Brodribb T. J. Cochard H. (2009). Hydraulic failure defines the recovery and point of death in water-stressed conifers. *Plant Physiol.* 149 575–584. 10.1104/pp.108.129783 19011001 PMC2613726

[B6] Bueso E. Rodriguez L. Lorenzo-Orts L. Gonzalez-Guzman M. Sayas E. Muñoz-Bertomeu J. (2014). The single-subunit RING-type E3 ubiquitin ligase RSL1 targets PYL4 and PYR1 ABA receptors in plasma membrane to modulate abscisic acid signaling. *Plant J.* 80 1057–1071. 10.1111/tpj.12708 25330042

[B7] Cao Y. Zhang Z. W. Xue L. W. Du J. B. Shang J. Xu F. (2009). Lack of salicylic acid in *Arabidopsis* protects plants against moderate salt stress. *Z. Naturforsch. C* 64 231–238. 10.1515/znc-2009-3-414 19526718

[B8] Carlberg I. Mannervik B. (1985). Glutathione reductase. *Methods Enzymol.* 113 484–490. 10.1016/S0076-6879(85)13062-4 3003504

[B9] Chance M. Maehly A. C. (1955). The assay of catalases and peroxidases. *Methods Enzymol.* 2 764–817. 10.1016/S0076-6879(55)02300-8 13193536

[B10] Chang Y. Nguyen B. H. Xie Y. Xiao B. Tang N. Zhu W. (2017). Co-overexpression of the constitutively active form of OsbZIP46 and ABA-activated protein kinase SAPK6 improves drought and temperature stress resistance in rice. *Front. Plant Sci.* 8:1102. 10.3389/fpls.2017.01102 28694815 PMC5483469

[B11] Contreras-Porcia L. Thomas D. Flores V. Correa J. A. (2011). Tolerance to oxidative stress induced by desiccation in *Porphyra columbina* (Bangiales, Rhodophyta). *J. Exp. Bot.* 62 1815–1829. 10.1093/jxb/erq364 21196477 PMC3060672

[B12] Doty S. L. Sher A. W. Fleck N. D. Khorasani M. Bumgarner R. E. Khan Z. (2016). Variable nitrogen fixation in wild *Populus*. *PLOS ONE* 11:e0155979. 10.1371/journal.pone.0155979 27196608 PMC4873266

[B13] Finkelstein R. R. Gampala S. S. Rock C. D. (2002). Abscisic acid signaling in seeds and seedlings. *Plant Cell* 14(Suppl.) S15–S45. 10.1105/tpc.010441 12045268 PMC151246

[B14] Fujii H. Verslues P. E. Zhu J. K. (2007). Identification of two protein kinases required for abscisic acid regulation of seed germination, root growth, and gene expression in *Arabidopsis*. *Plant Cell* 19 485–494. 10.1105/tpc.106.048538 17307925 PMC1867333

[B15] Giannopolitis C. N. Ries S. K. (1977). Superoxide dismutases occurrence in higher plants. *Plant Physiol.* 59 309–314. 10.1104/pp.59.2.309 16659839 PMC542387

[B16] Gill S. S. Tuteja N. (2010). Reactive oxygen species and antioxidant machinery in abiotic stress tolerance in crop plants. *Plant Physiol. Biochem.* 48 909–930. 10.1016/j.plaphy.2010.08.016 20870416

[B17] Gonzalez-Guzman M. Pizzio G. A. Antoni R. Vera-Sirera F. Merilo E. Bassel G. W. (2012). *Arabidopsis* PYR/PYL/RCAR receptors play a major role in quantitative regulation of stomatal aperture and transcriptional response to abscisic acid. *Plant Cell* 24 2483–2496. 10.1105/tpc.112.098574 22739828 PMC3406898

[B18] González-Guzmán M. Rodríguez L. Lorenzo-Orts L. Pons C. Sarrión-Perdigones A. Fernández M. A. (2014). Tomato PYR/PYL/RCAR abscisic acid receptors show high expression in root, differential sensitivity to the abscisic acid agonist quinabactin, and the capability to enhance plant drought resistance. *J. Exp. Bot.* 65 4451–4464. 10.1093/jxb/eru219 24863435 PMC4112642

[B19] Heath R. L. Packer L. (1968). Photoperoxidation in isolated chloroplasts. I. Kinetics and stoichiometry of fatty acid peroxidation. *Arch. Biochem. Biophys.* 125 189–198. 10.1016/0003-9861(68)90654-1 5655425

[B20] Himmelbach A. Yang Y. Grill E. (2003). Relay and control of abscisic acid signaling. *Curr. Opin. Plant Biol.* 6 470–479. 10.1016/S1369-5266(03)00090-6 12972048

[B21] Hu L. Li H. Pang H. Fu J. (2012). Responses of antioxidant gene, protein and enzymes to salinity stress in two genotypes of perennial ryegrass (*Lolium perenne*) differing in salt tolerance. *J. Plant Physiol.* 169 146–156. 10.1016/j.jplph.2011.08.020 22088275

[B22] Irigoyen M. L. Iniesto E. Rodriguez L. Puga M. I. Yanagawa Y. Pick E. (2014). Targeted degradation of abscisic acid receptors is mediated by the ubiquitin ligase substrate adaptor DDA1 in *Arabidopsis*. *Plant Cell* 26 712–728. 10.1105/tpc.113.122234 24563205 PMC3967035

[B23] Jefferson R. A. Kavanagh T. A. Bevan M. W. (1987). GUS fusions: beta-glucuronidase as a sensitive and versatile gene fusion marker in higher plants. *EMBO J.* 6 3901–3907. 3327686 10.1002/j.1460-2075.1987.tb02730.x PMC553867

[B24] Jiang M. Zhang J. (2002). Role of abscissic acid in water stress-induced antioxidant defense in leaves of maize seedlings. *Free Radic. Res.* 36 1001–1015. 10.1080/1071576021000006563 12448826

[B25] Kim H. Lee K. Hwang H. Bhatnagar N. Kim D. Y. Yoon I. S. (2014). Overexpression of *PYL5* in rice enhances drought tolerance, inhibits growth, and modulates gene expression. *J. Exp. Bot.* 65 453–464. 10.1093/jxb/ert397 24474809 PMC3904710

[B26] Knight H. Zarka D. G. Okamoto H. Thomashow M. F. Knight M. R. (2004). Abscisic acid induces *CBF* gene transcription and subsequent induction of cold-regulated genes via the *CRT* promoter element. *Plant Physiol.* 135 1710–1717. 10.1104/pp.104.043562 15247382 PMC519084

[B27] Kwak J. M. Mäser P. Schroeder J. I. (2008). The clickable guard cell, version II: interactive model of guard cell signal transduction mechanisms and pathways. *Arabidopsis Book* 6:e0114. 10.1199/tab.0114 22303239 PMC3243356

[B28] Lackman P. González-Guzmán M. Tilleman S. Carqueijeiro I. Pérez A. C. Moses T. (2011). Jasmonate signaling involves the abscisic acid receptor *PYL4* to regulate metabolic reprogramming in *Arabidopsis* and tobacco. *Proc. Natl. Acad. Sci. U.S.A.* 108 5891–5896. 10.1073/pnas.1103010108 21436041 PMC3078376

[B29] Lee S. C. Lan W. Z. Buchanan B. B. Luan S. (2009). A protein kinase-phosphatase pair interacts with an ion channel to regulate ABA signaling in plant guard cells. *Proc. Natl. Acad. Sci. U.S.A.* 106 21419–21424. 10.1073/pnas.0910601106 19955427 PMC2795491

[B30] Leung J. Giraudat J. (1998). Abscisic acid signal transduction. *Annu. Rev. Plant Physiol Plant Mol. Biol.* 49 199–222. 10.1146/annurev.arplant.49.1.199 15012233

[B31] Liu C. C. Liu Y. G. Guo K. Fan D. Y. Li G. Q. Zheng Y. R. (2011). Effect of drought on pigments, osmotic adjustment and antioxidant enzymes in six woody plant species in karst habitats of southwestern China. *Environ. Exp. Bot.* 71 174–183. 10.1016/j.envexpbot.2010.11.012

[B32] Liu X. Yue Y. Li B. Nie Y. Li W. Wu W. H. (2007). A G protein-coupled receptor is a plasma membrane receptor for the plant hormone abscisic acid. *Science* 315 1712–1716. 10.1126/science.1135882 17347412

[B33] Liu Y. Pan Z. Zhuang Q. Miralles D. G. Teuling A. J. Zhang T. (2015). Agriculture intensifies soil moisture decline in Northern China. *Sci. Rep.* 5:11261. 10.1038/srep11261 26158774 PMC4497304

[B34] Ma Y. Szostkiewicz I. Korte A. Moes D. Yang Y. Christmann A. (2009). Regulators of PP2C phosphatase activity function as abscisic acid sensors. *Science* 324 1064–1068. 10.1126/science.1172408 19407143

[B35] Maehly A. C. Chance B. (1954). The assay of catalases and per regulators of PP2C phosphatase activity function as abscisic acid sensors oxidases. *Methods Biochem. Anal.* 1 357–424. 13193536 10.1002/9780470110171.ch14

[B36] Maruyama K. Urano K. Yoshiwara K. Morishita Y. Sakurai N. Suzuki H. (2014). Integrated analysis of the effects of cold and dehydration on rice metabolites, phytohormones, and gene transcripts. *Plant Physiol.* 164 1759–1771. 10.1104/pp.113.231720 24515831 PMC3982739

[B37] Menconi M. Sgherri C. L. M. Pinzino C. Navari-Izzo F. (1995). Activated oxygen production and detoxification in wheat plants subjected to a water deficit programme. *J. Exp. Bot.* 46 1123–1130. 10.1093/jxb/46.9.1123

[B38] Mustilli A. C. Merlot S. Vavasseur A. Fenzi F. Giraudat J. (2002). Arabidopsis OST1 protein kinase mediates the regulation of stomatal aperture by abscisic acid and acts upstream of reactive oxygen species production. *Plant Cell* 14 3089–3099. 10.1105/tpc.007906 12468729 PMC151204

[B39] Nakano Y. Asada K. (1981). Hydrogen peroxide is scavenged by ascorbate-specific peroxidase in spinach chloroplasts. *Plant Cell Physiol.* 22 867–880.

[B40] Pandey S. Nelson D. C. Assmann S. M. (2009). Two novel GPCR-type G proteins are abscisic acid receptors in *Arabidopsis*. *Cell* 136 136–148. 10.1016/j.cell.2008.12.026 19135895

[B41] Park S. Y. Fung P. Nishimura N. Jensen D. R. Fujii H. Zhao Y. (2009). Abscisic acid inhibits type 2C protein phosphatases via the PYR/PYL family of START proteins. *Science* 324 1068–1071. 10.1126/science.1173041 19407142 PMC2827199

[B42] Petrov V. Hille J. Mueller-Rueber B. Gechev T. (2015). ROS-mediated abiotic-stress induced programmed cell death in plants. *Front. Plant Sci.* 6:69. 10.3389/fpls.2015.00069 25741354 PMC4332301

[B43] Pizzio G. A. Rodriguez L. Antoni R. Gonzalez-Guzman M. Yunta C. Merilo E. (2013). The PYL4 A194T mutant uncovers a key role of PYR1-LIKE4/PROTEIN PHOSPHATASE 2CA interaction for abscisic acid signaling and plant drought resistance. *Plant Physiol.* 163 441–455. 10.1104/pp.113.224162 23864556 PMC3762663

[B44] Saavedra X. Modrego A. Rodríguez D. González-García M. P. Sanz L. Nicolás G. (2010). The nuclear interactor PYL8/RCAR3 of Fagus sylvatica FsPP2C1 is a positive regulator of abscisic acid signaling in seeds and stress. *Plant Physiol.* 152 133–150. 10.1104/pp.109.146381 19889877 PMC2799352

[B45] Santiago J. Rodrigues A. Saez A. Rubio S. Antoni R. Dupeux F. (2009). Modulation of drought resistance by the abscisic acid receptor PYL5 through inhibition of clade A PP2Cs. *Plant J.* 60 575–588. 10.1111/j.1365-313X.2009.03981.x 19624469

[B46] Seki M. Ishida J. Narusaka M. Fujita M. Nanjo T. Umezawa T. (2002). Monitoring the expression pattern of around 7,000 *Arabidopsis* genes under ABA treatments using a foil-length cDNA microarray. *Funct. Integr. Genomics* 2 282–291. 10.1007/s10142-002-0070-6 12444421

[B47] Shanker A. K. Maheswari M. Yadav S. K. Desai S. Bhanu D. Attal N. B. (2014). Drought stress responses in crops. *Funct. Integr. Genomics* 14 11–22. 10.1007/s10142-013-0356-x 24408129

[B48] Shen Y. Y. Wang X. F. Wu F. Q. Du S. Y. Cao Z. Shang Y. (2006). The Mg-chelatase H subunit is an abscisic acid receptor. *Nature* 443 823–826. 10.1038/nature05176 17051210

[B49] Sivakumar M. V. K. Das H. P. Brunini O. (2005). Impacts of present and future climate variability and change on agriculture and forestry in the arid and semi-arid tropics. *Clim. Change* 70 31–72. 10.1007/s10584-005-5937-9

[B50] Skirycz A. Inzé D. (2010). More from less: plant growth under limited water. *Curr. Opin. Biotechnol.* 21 197–203. 10.1016/j.copbio.2010.03.002 20363612

[B51] Sochor J. Ruttkay-Nedecky B. Babula P. Adam V. Hubalek J. Kizek R. (2012). “Automation of methods for determination of lipid peroxidation,” in *Lipid Peroxidation* ed. Catala A. (Rijeka: In Tech).

[B52] Souza R. P. Machado E. C. Silva J. A. B. Lagôa A. M. M. A. Silveira J. A. G. (2004). Photosynthetic gas exchange, chlorophyll fluorescence and some associated metabolic changes in cowpea (*Vigna unguiculata*) during water stress and recovery. *Environ. Exp. Bot.* 51 45–56. 10.1016/S0098-8472(03)00059-5

[B53] Štajner D. Orlovic S. Popovic B. Kebert M. Galic Z. (2011). Screening of drought oxidative stress tolerance in Serbian melliferous plant species. *Afr. J. Biotechnol.* 10 1609–1614.

[B54] Suzuki N. Koussevitzky S. Mittler R. Miller G. (2012). ROS and redox signaling in the response of plants to abiotic stress. *Plant Cell Environ.* 35 259–270. 10.1111/j.1365-3040.2011.02336.x 21486305

[B55] Tian X. Wang Z. Li X. Lv T. Liu H. Wang L. (2015). Characterization and functional analysis of pyrabactin resistance-like abscisic acid receptor family in rice. *Rice* 8 28. 10.1186/s12284-015-0061-6 26362328 PMC4567572

[B56] Tyree M. T. Sperry J. S. (1988). Do woody plants operate near the point of catastrophic xylem dysfunction caused by dynamic water stress? Answers from a model. *Plant Physiol.* 88 547–580. 10.1104/pp.88.3.574 16666351 PMC1055627

[B57] Wang J. Wu B. Yin H. Fan Z. Li X. Ni S. (2017). Overexpression of *CaAPX* induces orchestrated reactive oxygen scavenging and enhances cold and heat tolerances in tobacco. *Biomed Res. Int.* 2017:4049534. 10.1155/2017/4049534 28386551 PMC5366785

[B58] Wu Z. G. Jiang W. Chen S. L. Mantri N. Tao Z. M. Jiang C. X. (2016). Insights from the cold transcriptome and metabolome of *Dendrobium officinale*: global reprogramming of metabolic and gene regulation networks during cold acclimation. *Front. Plant Sci.* 7:1653. 10.3389/fpls.2016.01653 27877182 PMC5099257

[B59] Yang Y. Qi M. Mei C. (2004). Endogenous salicylic acid protects rice plants from oxidative damage caused by aging as well as biotic and abiotic stress. *Plant J.* 40 909–919. 10.1111/j.1365-313X.2004.02267.x 15584956

[B60] Yoshida R. Hobo T. Ichimura K. Mizoguchi T. Takahashi F. Aronso J. (2002). ABA-activated SnRK2 protein kinase is required for dehydration stress signaling in *Arabidopsis*. *Plant Cell Physiol.* 43 1473–1483. 10.1093/pcp/pcf188 12514244

[B61] Yoshimura K. Miyao K. Gaber A. Takeda T. Kanaboshi H. Miyasaka H. (2004). Enhancement of stress tolerance in transgenic tobacco plants overexpressing *Chlamydomonas* glutathione peroxidase in chloroplasts or cytosol. *Plant J.* 37 21–33. 10.1046/j.1365-313X.2003.01930.x 14675429

[B62] Yu J. L. Yang L. Liu X. Tang R. J. Wang Y. Ge H. M. (2016). Overexpression of poplar pyrabactin resistance-like abscisic acid receptors promotes abscisic acid sensitivity and drought resistance in transgenic *Arabidopsis*. *PLOS ONE* 11:e0168040. 10.1371/journal.pone.0168040 27992471 PMC5167274

[B63] Zeevaart J. A. D. Creelman R. A. (1998). Metabolism and physiology of abscisic acid. *Annu. Rev. Plant Physiol. Plant Mol. Biol.* 39 439–473. 10.1146/annurev.pp.39.060188.002255

[B64] Zhao Y. Chan Z. Gao J. Xing L. Cao M. Yu C. (2016). ABA receptor PYL9 promotes drought resistance and leaf senescence. *Proc. Natl. Acad. Sci. U.S.A.* 113 1949–1954. 10.1073/pnas.1522840113 26831097 PMC4763734

[B65] Zhou L. Wang S. Chi Y. Li Q. Huang K. Yu Q. (2015). Responses of photosynthetic parameters to drought in subtropical forest ecosystem of China. *Sci. Rep.* 5:18254. 10.1038/srep18254 26666469 PMC4678887

[B66] Zhu J. K. (2002). Salt and drought stress signal transduction in plants. *Annu. Rev. Plant Biol.* 53 247–273. 10.1146/annurev.arplant.53.091401.143329 12221975 PMC3128348

