# Supplementary figures and images for: Overexpression of Pyrabactin Resistance-Like Abscisic Acid Receptors Enhances Drought, Osmotic, and Cold Tolerance in Transgenic Poplars
Source: Front Plant Sci. 2017 Oct 13;8:1752. doi: 10.3389/fpls.2017.01752 (PMC5645508; doi:10.3389/fpls.2017.01752)

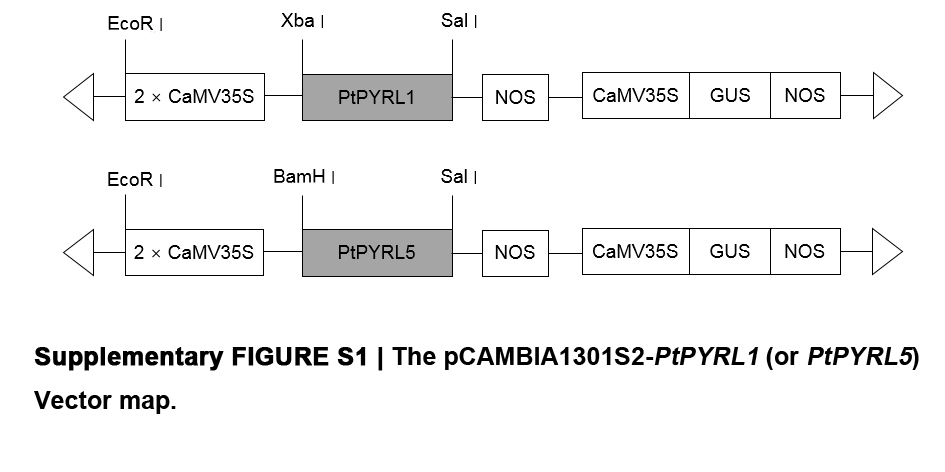

Supplement: FIGURE S1 — The pCAMBIA1301S2-PtPYRL1 (or PtPYRL5) Vector map. [file Image_1.JPEG]

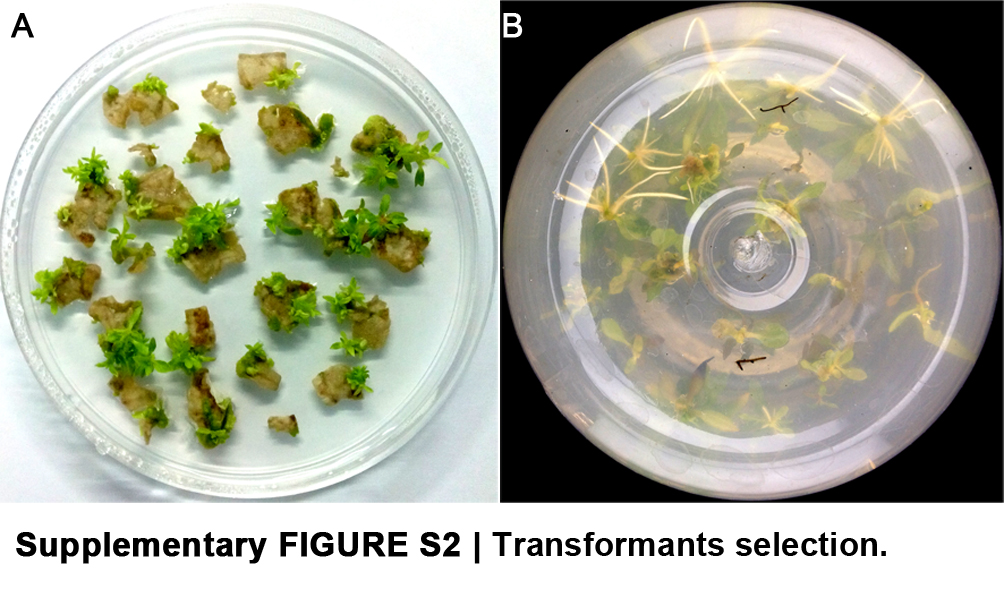

Supplement: FIGURE S2 — Transformants selection. (A) Adventitious bud regenerated from selection medium. (B) Transgenic shoots formed roots on selection medium. [file Image_2.JPEG]

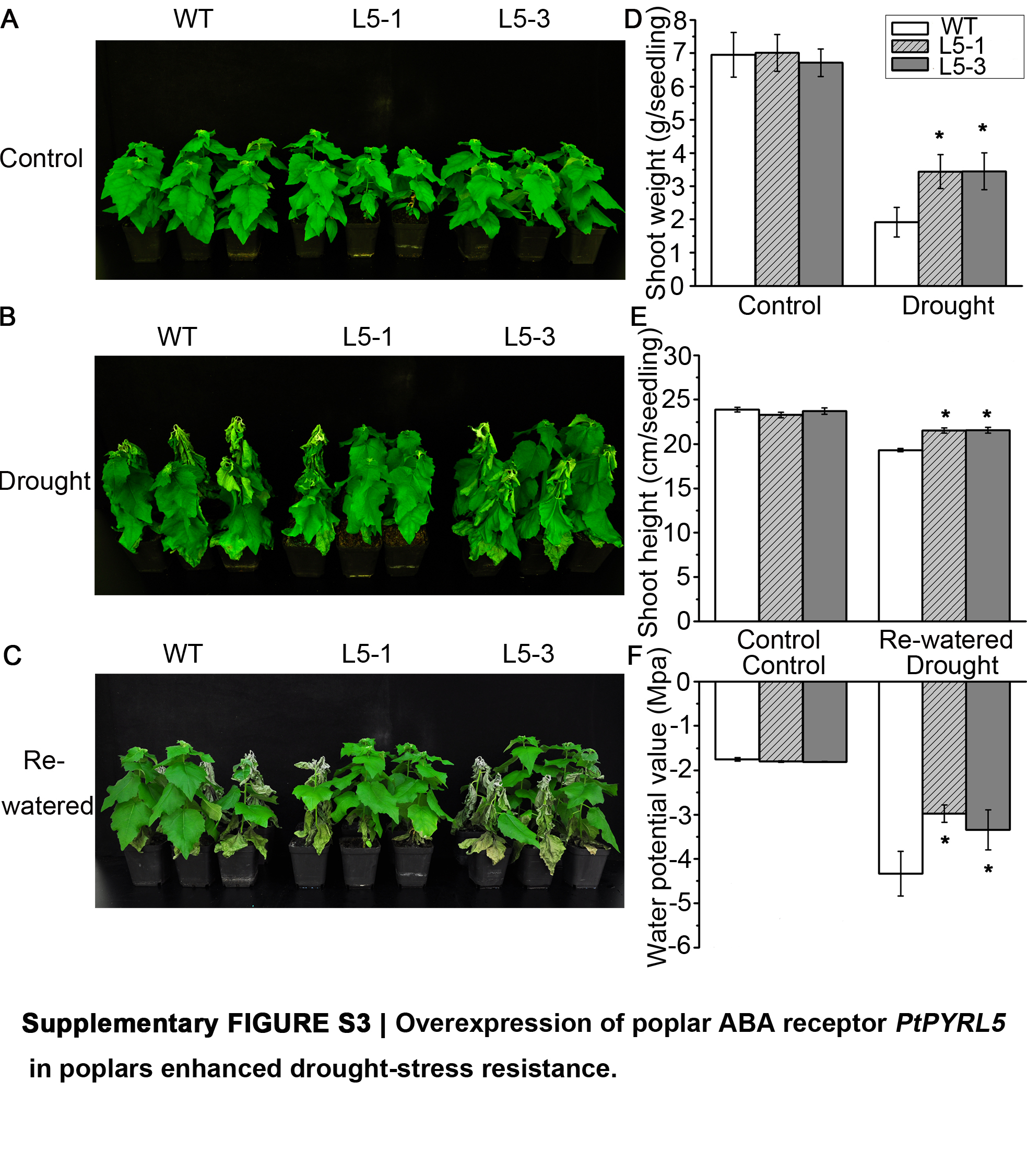

Supplement: FIGURE S3 — Overexpression of poplar ABA receptor PtPYRL5 in poplars enhanced drought-stress resistance. (A) 2-month-old WT (non-transgenic hybrid poplars) and transgenic poplars (L5-1 and L5-3) were cultured in the greenhouse with normal watering. (B) WT and transgenic hybrid poplars were not watered for 5 days. (C) After drought, WT and transgenic hybrid poplars were then re-watered for 3 days. The shoot weight after drought stress treatment (D), shoot height after re-watered for 3 days (E), water potential value (F) of WT and transgenic hybrid poplars were measured. Values are means ± SD (one-way ANOVA test; ∗P < 0.05 as compared to WT). [file Image_3.JPEG]

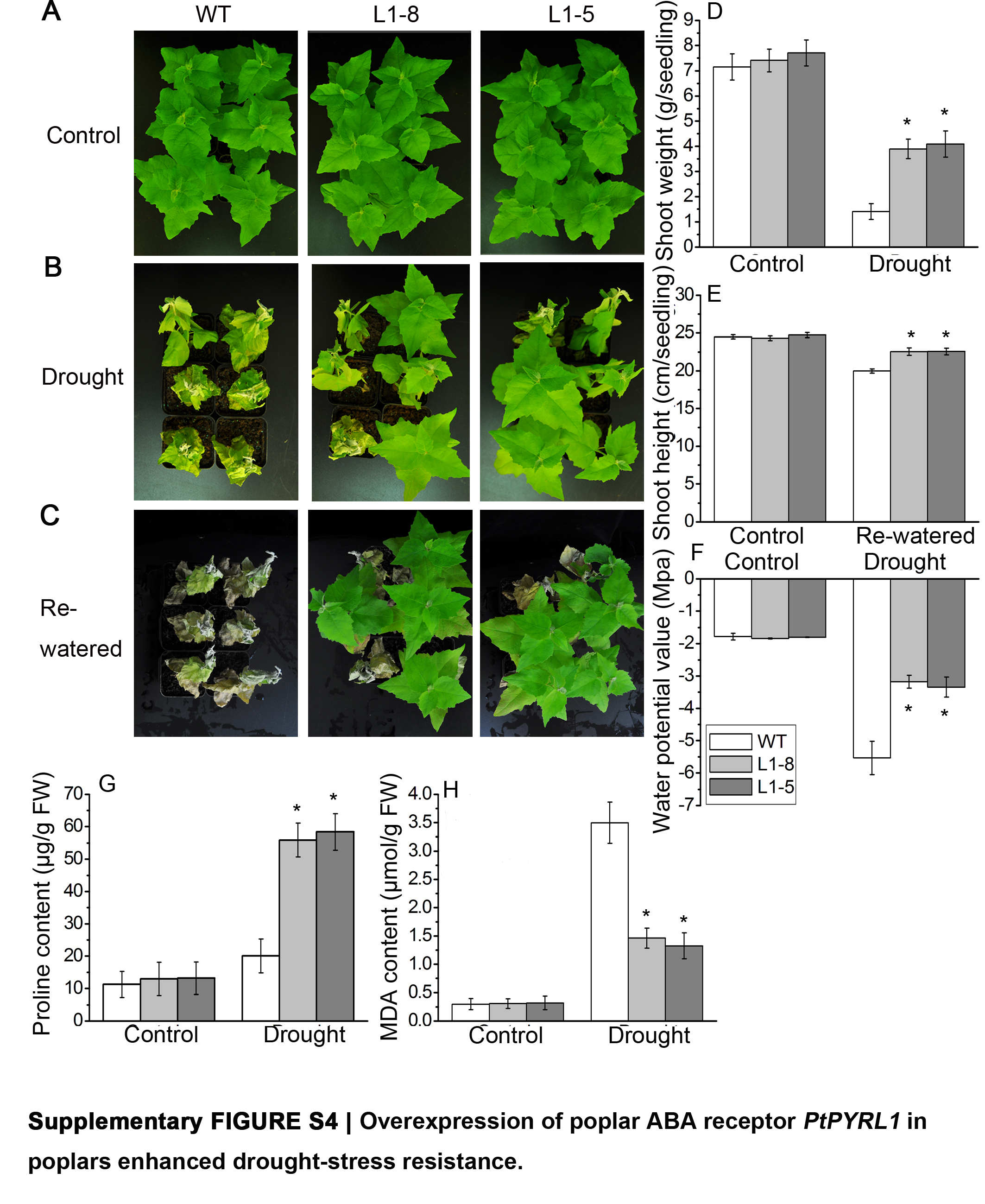

Supplement: FIGURE S4 — Overexpression of poplar ABA receptor PtPYRL1 in poplars enhanced drought-stress resistance. (A) 2-month-old WT (non-transgenic hybrid poplars) and transgenic poplars (L1-8 and L1-5) were cultured in the greenhouse with normal watering. (B) WT and transgenic hybrid poplars were not watered for 5 days. (C) After drought, WT and transgenic hybrid poplars were then re-watered for 3 days. The shoot weight after drought stress treatment (D), shoot height after re-watered for 3 days (E), water potential value (F), proline content (G) and MDA content (H) of WT and transgenic hybrid poplars were measured. Values are means ± SD (n = 18, three independent experiments, one-way ANOVA test; ∗P < 0.05). [file Image_4.JPEG]
