# Supplementary material for: Overexpression of Pyrabactin Resistance-Like Abscisic Acid Receptors Enhances Drought, Osmotic, and Cold Tolerance in Transgenic Poplars
Source: Front Plant Sci. 2017 Oct 13;8:1752. doi: 10.3389/fpls.2017.01752 (PMC5645508; doi:10.3389/fpls.2017.01752)
Supplement: Supplementary file 5 [file Table_1.docx]

**Supplementary Table S1. List of primers used in this study**.

| **Names** | **Sequences (5’ to 3’)** | **Purposes** |
| --- | --- | --- |
| *PtPYRL1*-RT-F | CCCACCACGTCACAATCCCACCTAG | *PtPYRL1* qRT-PCR |
| *PtPYRL1*-RT-R | GCCACGCTGCAGCTCTTGATGAAG |  |
| *PtPYRL5*-RT-F | GCCGCCATCCCTACCACAACAAC | *PtPYRL5* qRT-PCR |
| *PtPYRL5*-RT-R | CGGTGGAGACTGGTGCATTGATGG |  |
| *EF1β*-RT-F | GACAAGAAGGCAGCGGAGGAGAG | *EF1β* qRT-PCR |
| *EF1β*-RT-R | CAATGAGGGAATCCACTGACACAAG |  |
| P35S | TGACGCACAATCCCACTATC | General Use |
| *PtPYRL1*-F | GGGTCTAGAATGACTGACCCAGCACAACAAGAAC | *PtPYRL1* genomic PCR |
| *PtPYRL1*-R | GGGGTCGACTTATTTACCGTCACCGTCACGAGC |  |
| *PtPYRL5*-F | GGGGGATCCATGCCTGCATCACTACAGCTCCAG | *PtPYRL5* genomic PCR |
| *PtPYRL5*-R | GGGGTCGACTCATGATGATGTAGAAATCTGGGCAT |  |
